# Fitness Costs of Chlorantraniliprole Resistance Related to the *SeNPF* Overexpression in the *Spodoptera exigua* (Lepidoptera: Noctuidae)

**DOI:** 10.3390/ijms22095027

**Published:** 2021-05-10

**Authors:** Changwei Gong, Xinge Yao, Qunfang Yang, Xuegui Wang, Yuming Zhang, Yumeng Wang, Litao Shen

**Affiliations:** Biorational Pesticide Research Laboratory, Agricultural College, Sichuan Agricultural University, Chengdu 611130, China; youguqiu@163.com (C.G.); yaoxinge@sicau.edu.cn (X.Y.); lmk94811@163.com (Q.Y.); m18408215896@163.com (Y.Z.); 18328695032@139.com (Y.W.); shen-litao@163.com (L.S.)

**Keywords:** ryanodine receptors, vitellogenin, post-mating response, mating rate, oviposition

## Abstract

*Spodopteraexigua*, a multifeeding insect pest, has developed a high level of resistance to chlorantraniliprole, which is a benzoylurea insecticide that targets the ryanodine receptors (RyRs). Herein, the resistant strain (SE-Sel) and sensitive strain (SE-Sus) were obtained by bidirectional screening for six generations. The potential oviposited eggs and oviposition rate of the SE-Sel strain were dramatically lower than those of the SE-Sus strain; on the contrary, the weights of prepupae and preadult were significantly increased. As a post-mating response, the higher number of non-oviposited eggs in the SE-Sel strain was caused by a lower mating rate. In addition, the expression levels of *vitellogenin* (*SeVg*) and its receptor (*SeVgR*) in the SE-Sel strain were consistently lower than those in the SE-Sus strain. An RyR^I4743M^ mutation, contributing to the resistance to chlorantraniliprole, was located in the S3 transmembrane segments and might have affected the release of calcium ions; it led to the upregulated expression of the neuropeptide *SeNPF* and its receptor *SeNPFR*, and the mating and oviposition rate were significantly recovered when the *SeNPF* was knocked down though RNA interference (RNAi) in the male adult of the SE-Sel strain. Moreover, the expression of the juvenile hormone-binding proteins *SeJHBWDS3* and *SeJHBAN* in the male adult of the SE-Sel strain was significantly decreased, which proved the existence of a fitness cost from another angle. Therefore, these results indicate that the fitness cost accompanied by chlorantraniliprole resistance in *S. exigua* may be related to the decrease in mating desire due to *SeNPF* overexpression.

## 1. Introduction

The beet armyworm, *Spodoptera exigua* Hübner, an important insect pest that threatens numerous cash crops, such as *Gossypium hirsutum*, *Zea mays*, *Nicotiana tabacum*, and *Medicago sativa*, survives wintering by migration without diapause and causes economic losses worldwide [1,2,3,4]. Chemical insecticides have been the most effective means of controlling this pest for the last two decades, including some traditional organophosphorus, pyrethroid, and benzoylurea insecticides and other supplemented newer insecticides in recent years [5,6,7,8,9].

Chlorantraniliprole is a novel benzoylurea insecticide that targets the ryanodine receptors (RyRs), which are ion channels that regulate intracellular Ca^2+^ release during physiological excitation–contraction coupling [10], leads to the over-release of internal calcium ion (Ca^2+^) stores, and subsequently causes feeding cessation, muscle paralysis, and ultimately death [11,12], and the over-release of Ca^2+^ also enhances neuropeptide activation to regulate ovarian development [10,13,14]. It is effective against almost all lepidopteran pests and is safe for mammals, birds, and fishes [15,16]. However, owing to the overuse of this insecticide, field populations collected from different districts in China have evolved a high level of resistance to chlorantraniliprole [6,17]. In 2012, 16 populations of *S. exigua* in seven provinces in China were monitored for their resistance levels, with resistance ratios (RR) of 2-44-fold to chlorantraniliprole [7]. In recent years, Guo et al. [18] also declared their discovery of a field-resistant population of *Plutella xylostella* with an RR of 2128 to chlorantraniliprole in Yunnan Province. Wang et al. [19] reported that the resistance of *P. xylostella* to chlorantraniliprole in Guangdong Province had reached 2000-fold. Wang et al. [20] found that the resistances of *S. exigua* collected in 2016 were significantly higher than two years earlier, especially for chlorantraniliprole, with RRs rising from 173.4–to 582.6-fold.

Normally, the evolution of resistance to an insecticide is accompanied by a high cost or striking weaknesses that could diminish the insect’s fitness in comparison to its susceptible counterparts in the population [21,22], especially for reproduction fitness costs; for example, resistant insects have lower fertility and longer developmental duration than sensitive insects, which is not conducive to insect population growth. Cao and Han [23] found that the mating rate and hatching rate of a tebufenozide-resistant *P. xylostella* strain were significantly reduced. Ribeiro et al. [24] found that the chlorantraniliprole-resistant strain of *P. xylostella* presented significantly lower fecundity and higher larval and pupal periods, hatchability, and male longevity compared with the susceptible strain. Abbas et al. [25] also found that profenofos-resistant strains of *Spodoptera litura* have reduced fertility and hatching rates and lower intrinsic growth rates. The resistant strain has shown an adaptive cost in comparison to the susceptible strain, which could result in a delay in population growth in the field [26].

Vitellogenin (Vg) is an indispensable reproduction-related protein that is traditionally identified as an appropriate parameter for appraising female fertility in insects [27]. In most insects, Vg is mainly synthesized by fat bodies or other tissues—for example, ovarian tissues [28]—secreted to the hemolymph, binds to the vitellogenin receptor, and finally enters the cell through endocytosis after reaching the oocyte’s surface [29,30]. In our laboratory, a resistant strain (SE-Sel) of *S. exigua* was established through continuous selection with a sublethal dose of chlorantraniliprole, and a fitness cost accompanied with chlorantraniliprole resistance was found. However, the resistance mechanism and fitness cost accompanied by the insecticide were still not clear. Identifying the fitness cost and the dominance of the fitness cost accompanied by insecticide resistance could be useful for designing an integrated pest management (IPM) program that limits the rapid propagation of the resistant population [22]. Therefore, in the current study, a comparison of the ability and gonad coefficiency between the SE-Sel and sensitive strains (SE-Sus) was employed to investigate whether the fitness cost was accompanied with chlorantraniliprole resistance in *S. exigua*. In addition, transcriptome analysis of the SE-Sel and SE-Sus strains, and reproduction-related genes, was performed to investigate the underlying mechanism. The results of the present study can be useful in designing appropriate management strategies for resistant insect populations.

## 2. Results

### 2.1. Comparison of the Reproductive Capacity and Egg Hatchability between the SE-Sus and SE-Sel Strains

**Figure 1:**
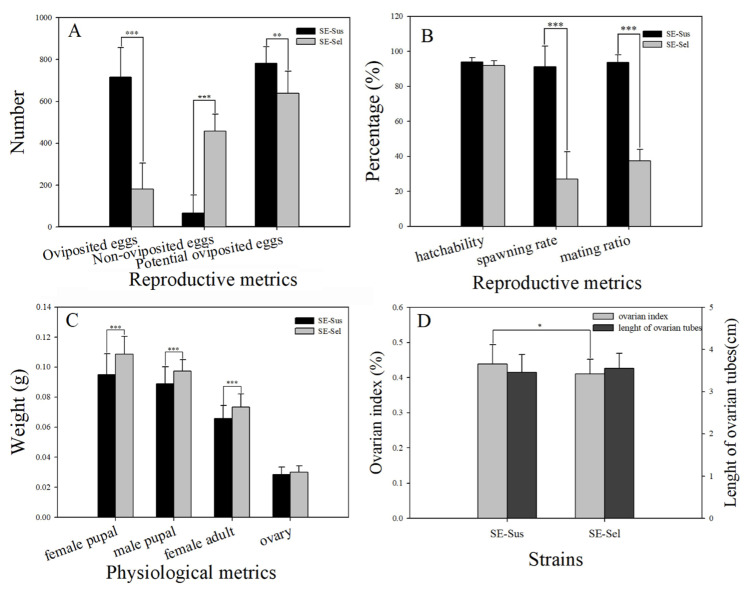
The comparison of reproductive metrics and physiological metrics between SE-Sus and SE-Sel strains. (**A**) The numbers of oviposited eggs, non-oviposited eggs, and potential oviposited eggs; (**B**) The hatchability, spawning, and mating rate; (**C**) The weight of female pupal, male pupal, female adult, and ovary; (**D**) The ovarian index and length of ovarian tubes. Different numbers of asterisks indicate significant differences at different levels (* means *p* < 0.05, ** means *p* < 0.01, *** means *p* < 0.001).

In order to research the fitness of resistant strains, the reproductive capacity and egg hatchability between the SE-Sus and SE-Sel strains were compared. The average amount of oviposited eggs per female of the SE-Sus strain was 715.50, which was significantly higher than that of the SE-Sel strain (181.79) (Figure 1A). After dissecting dead female adults, the average amount of non-oviposited eggs per female for the SE-Sus strain only reached 66.17 and was significantly lower than that in the SE-Sel strain (457.29) (Figure 1A). Therefore, the potential oviposited eggs and oviposition rates of the SE-Sus strain were 781.67 and 91.08%, respectively, which were significantly higher than those of the SE-Sel strain at 639.08 and 27.11% (Figure 1A,B). The mating rate of the SE-Sus strain (93.75%) was significantly higher than that of the SE-Sel strain (37.5%), but the gap in egg hatchability between SE-Sus and SE-Sel was only 2.16%, with no significant difference (Figure 1B).

### 2.2. Comparison of the Ovarian Index between the SE-Sus and SE-Sel Strains

Their weights, ovarian index, and the length of ovarian tubes were detected for verifying the fitness changes in the SE-Sus strain compared to those in the SE-Sel Strain. The mean pupal weights of each female and male of the SE-Sus strain were 0.0949 g and 0.089 g, respectively, which were significantly lower than those of the SE-Sel strain (0.1086 g and 0.0974 g, respectively) (Figure 1C). The mean weight of each female adult of the SE-Sus strain (0.0656 g) was significantly lower than that of the SE-Sel strain (0.0734 g); meanwhile, the weight of preovary and the length of ovarian tubes obtained from dissecting female adults of the SE-Sus strain (0.0286 g and 3.46 cm) were also lower than those of the SE-Sel strain (0.0300 g and 3.55 cm), even though there was no significant difference (*p* > 0.05) (Figure 1C); the ovarian index of female adults of the SE-Sus strain (0.4382) was significantly higher than that of the SE-Sel strain (0.4102) (Figure 1D).

### 2.3. Gene Expression Pattern of SeVg in the SE-Sus and SE-Sel Strains

**Figure 2:**
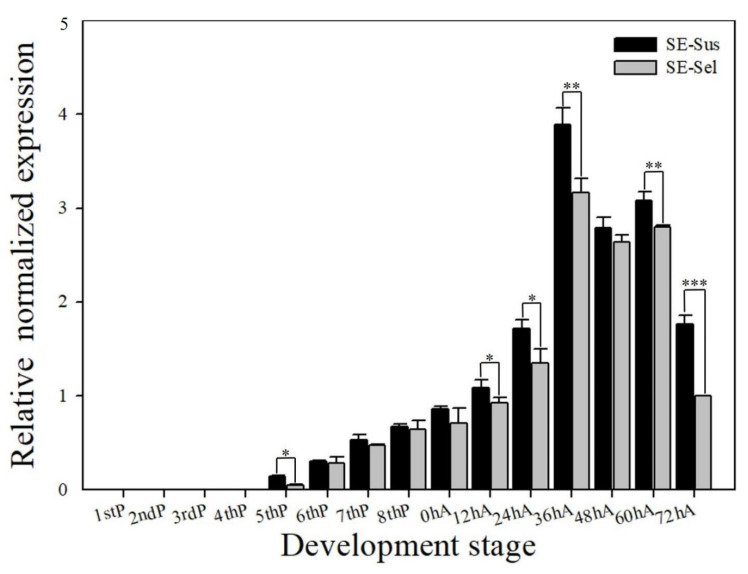
The relative normalized expression of the *SeVg gene* at different development stages of in the SE-Sus and SE-Sel strains. 1stP, 2ndP, 3rdP, 4thP, 5thP, 6thP, 7thP, and 8thP indicate 1 to 8 days after pupation, respectively. 0 hA, 12 hA, 24 hA, 36 hA, 48 hA, 60 hA, and 72 hA indicate 0 to 72 h after emergence, respectively. Different letters (a, b) above bars indicate significant differences (*p* < 0.05) according to Tukey’s multiple range test. Different numbers of asterisks indicate significant differences at different levels (* means *p* < 0.05, ** means *p* < 0.01, *** means *p* < 0.001).

To determine whether the fitness cost was related to ovarian development, the relative expression of the *SeVg* gene in SE-Sus and SE-Sel females was detected at different times, and the results indicated that the *SeVg* gene in the two strains began to be expressed on the fifth day of the pupal stage. With the elongation and emergence of pupae, the expression level of the *SeVg* gene firstly increased until 36 h after emergence, reached the highest value, then began to decline. At 60 h after emergence, the expression level reached the second highest value, and then decreased again. The expression of the *SeVg gen*e in the SE-Sus strain was significantly stronger than that in the SE-Sel strain (*p* < 0.05), except for the sixth and eight days of pupation and 0 and 48 h after emergence (Figure 2).

### 2.4. Illumina Sequencing, Read Assembly, and Annotation

The statistics of reads, its assembly, and annotation were compared to represent the transcriptome data (https://www.ncbi.nlm.nih.gov/bioproject/PRJNA587951, accessed on 7 May 2021) and explore the underlying mechanism of the fitness cost. The total number of reads (150 bp/read) obtained from the SE-Sus and SE-Sel strains assessed in triplicate was 296,690,624, with 42,072,370 reads at least for each sample. The proportion of reads including linker sequences achieved 0.42~0.47%, and that of low-quality reads, which included reads with > 10% Ns and an abased number of Q ≤ 10 in > 50% of the total reads, reached 1.45 ~ 1.54%. After the linker sequences or low-quality reads were filtered out, 290,810,118 clean reads were collected, and the Q20 and Q30 were more than 98.91 and 96.64%, respectively (Supplementary Table S1).

The clean reads constituted mixed pools and were spliced into approximately 39,957 unigenes, and the longest unigene contained 20,539 nt, but the shortest unigene contained 201 nt (Supplementary Figure S1A). The N50 value—that is, the cumulative fragmentary length that reaches 50% of the total fragmentary length—was 2161 nt. There were 11,513 unigenes between 200 and 299 nt in length and 3268 unigenes of over 3000 nt in length. Moreover, 4295 unigenes contained over 10,000 reads, while 17,179 unigenes were composed of only 11~100 reads (Supplementary Figure S1B).

In the Nr database, 18,474 unigenes were successfully obtained exegeses, and the species (top 4) with the greatest number of homologous genes were *Amyelois transitella* (3493), *Bombyx mori* (3234), *Papilio xuthus* (1850), and *Papilio machaon* (1426) (Supplementary Figure S2A). Additionally, 11,891 unigenes were annotated in the SwissProt database, 11,671 unigenes were annotated in the KOG database, 11,891 unigenes were annotated in the KEGG database, and 8020 unigenes were annotated in these four databases (Supplementary Figure S2B. Among the 11,671 unigenes annotated in the KOG database, 5024 unigenes were categorized as general function prediction only, accounting for the largest proportion, and 3388 unigenes were categorized as signal transduction mechanisms (Supplementary Figure S2C).

### 2.5. Analysis of Gene Expression and Cluster Analysis of DEGs

**Figure 3:**
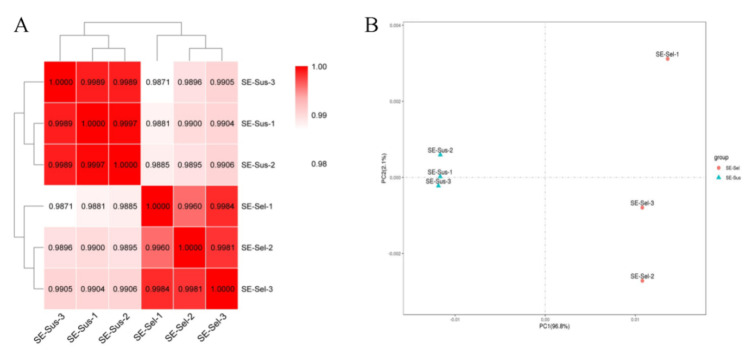
Heat map and principal component analysis (PCA) of gene expression levels in the six samples. (**A**) Heat map; (**B**) Principal component analysis (PCA). In Figure 4A, the darker the color is, the greater the correlation is.

The gene expression and cluster were analyzed to study their reproducibility-related genes obtained from the transcriptome. The correlations of gene expression levels in the SE-Sus strains (with a correlation index of 0.9989 to 1.0000) were dramatically higher than those in the SE-Sel strains (with a correlation index of 0.9871 to 0.9906) (Figure 3A). Additionally, there was a significant clustering relationship on PC1 between the samples in the SE-Sus strain and the SE-Sel strain, respectively, and the contribution degree of PC1 (96.8%) was dramatically higher than that of PC2 (2.1%) (Figure 3B).

**Figure 4:**
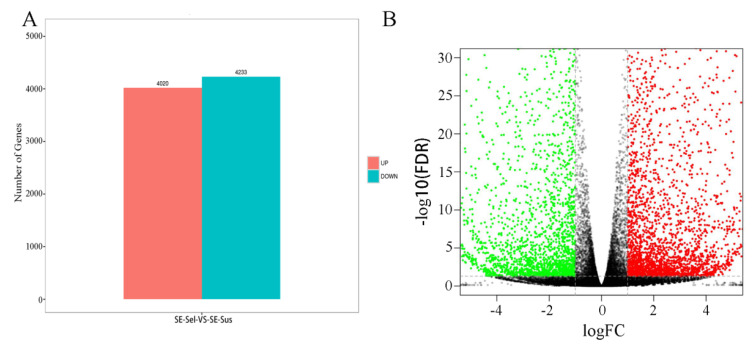
The statistical maps and volcano plot of DEGs among SE-Sel vs. SE-Sus. (**A**) The statistical maps; (**B**) The volcano plot. In Figure 5B, color-scaled represents log 2 (fold change) values for resistant lines, red indicates upregulated genes, green indicates dow-regulated genes, and black indicates genes with no difference in expression.

Under the screening threshold value of FDR < 0.05 and |log2FC| > 1, 8253 DEGs were obtained from the SE-Sel strain in comparison with the SE-Sus strain, 4020 of which were upregulated, while 4233 were downregulated (Figure 4A,B).

### 2.6. GO and KEGG Enrichment of DEGs

The annotation of DEGs was analyzed to enrich and screen possible candidate genes. The DEGs were enriched and assessed in the three Gene Ontology (GO) categories of biological process, cellular component, and molecular function. Among the DEGs enriched in molecular function, catalytic activity (GO: 0003824) was the most abundant (109), accounting for 72.19%, while in the cellular component category, cell (GO: 0005623) and cell part (GO: 0044464) were the most abundant (34.4%), and in the biological process category, metabolic process (GO: 0008152) was the most abundant (94), accounting for 65.73% (Supplementary Figure S3).

A total of 385 DEGs were enriched in the KEGG database and were related to metabolism, environmental information processing, and cellular processes. The enriched DEGs found in the KEGG database were> mainly in categories such as “biosynthesis of secondary metabolites” (with a *p*-value of 4.72 × 10^−6^), “neuroactive ligand–receptor interaction” (*p*-value of 1.82 × 10^−5^), “metabolic pathways” (*p*-value of 3.94 × 10^−5^), “lysosome” (*p*-value of 0.000223), “glycine, serine and threonine metabolism” (*p*-value of 0.000385), and “drug metabolism—other enzymes” *(p*-value of 0.00055). Additionally, the greatest number of unigenes enriched in metabolic pathways was 163 (Supplementary Figure S4).

### 2.7. Screening the Candidate Genes and Quantitative PCR (RT-qPCR)

In order to verify and further screen candidate genes, the candidate genes and their RT-qPCR were detected and analyzed. Compared with the SE-Sus strain, the SE-Sel strain exhibited 22 significantly up- or downregulated DEG unigenes, annotated as juvenile hormone-binding proteins, vitellogenin receptors, neuropeptides, and neuropeptide receptors (Supplementary Table S2).

**Figure 5:**
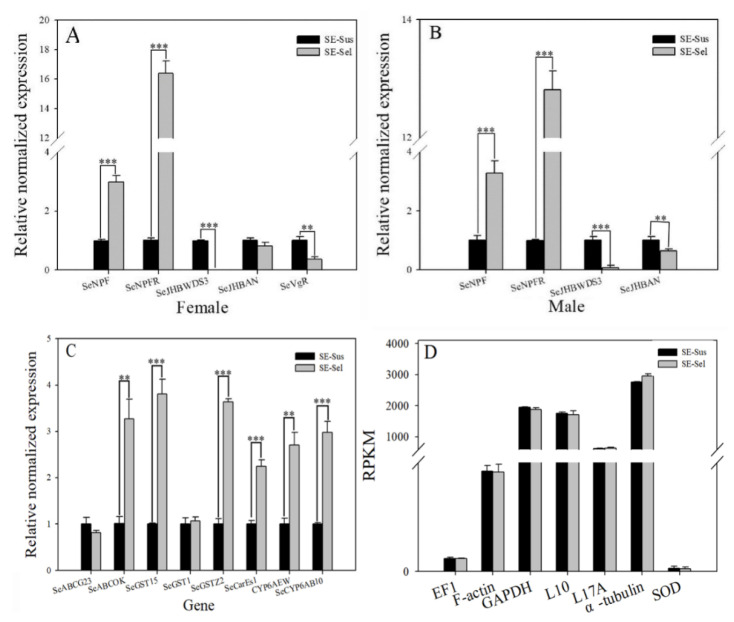
Gene expression pattern of the candidate genes in the SE-Sus and SE-Sel strains. (**A**) The relative normalized expression of *SeNPF*, *SeNPFR*, *SeJHBAN*, *SeJHBWDS3*, and *SeVgR* of female adult in the SE-Sus and SE-Sel strains; (**B**) The relative normalized expression of *SeNPF*, *SeNPFR*, *SeJHBAN*, and *SeJHBWDS3* of male adult; (**C**) The relative normalized expression of detoxification metabolism genes of adult; (**D**) The expression level (RPKM) of reference gene of adult. Different numbers of asterisks indicate significant differences at different levels (** means *p* < 0.01, *** means *p* < 0.001).

The results of RT-qPCR indicated that the relative normalized expression levels of *SeJHBWDS3* and *SeVgR* in the female adult of SE-Sel strain were significantly decreased by 0.01- and 0.36-fold (*p* < 0.05), whereas *SeNPF* and *SeNPFR* showed 4.33- and 16.39-fold increases, respectively, compared with the SE-Sus strain (*p* < 0.05). Additionally, the relative normalized expression levels of *SeJHBWDS3* and *SeJHBAN* in the male adult of SE-Sel strain were significantly decreased by 0.07- and 0.64-fold (*p* < 0.05), and *SeNPF* and *SeNPFR* increased by 3.27- and 12.81-fold. The results also indicated that the relative normalized expression levels of ABC transporter *(SeABCOK)*, glutathione *S-transferases (SeGST15* and *SeGSTZ2)*, carboxylesterases *(SeCarEs1)*, and cytochrome P450s (*CYP6AEW* and *SeCYP6AB10*) in the SE-Sel strain reached 3.27-, 3.81-, 3.64-, 2.25-, 2.70- and 2.98-fold, respectively, and were significantly higher than those in the SE-Sus strain (*p* < 0.05). However, there was no significant difference between two strains in the *elongation factor1 (EF1)*, *glyceralde hyde-3-phosphate dehydrogenase (GAPDH)*, *ribosomal protein L10 (L10)*, *ribosomal protein L17A (L17A)*, *superoxide dismutase (SOD)*, *α-tubulin (TUB)*, and *F-actin* (Figure 5).

### 2.8. Neuropeptide Diversity Analysis

**Figure 6:**
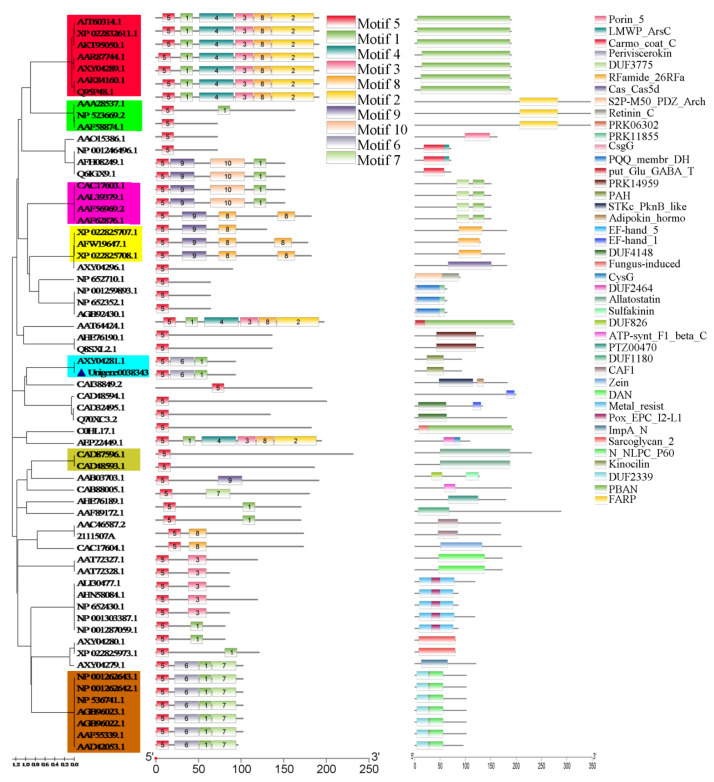
Phylogenetic tree of *neuropeptide* gene family constructed by NJ method and gene molecular structure map predicted by motif search and meme. In the phylogenetic tree, red-marked areas represent the PBAN family, green-marked areas represent the FMRF family, purple-marked areas represent the PBAN family with the adjunction of DUF3775 functional domains, brown-marked areas represent the neuropeptide F family, tan-marked areas represent the Allatostatin family, blue-marked areas represent the neuropeptide Y family.

To clarify the evolutionary conservation type of Neuropeptide in different insects and lay the foundation for subsequent structural model analysis, the structural and functional domains of Neuropeptide were analyzed by means of bioinformatics. Ten motifs (motif1~motif10), composed of highly conserved amino acid residues, were found in 63 neuropeptides amino acid sequences of *Drosophila melanogaster* and *Spodoptera* insects by meme search (http://meme-suite.org/tools/meme, accessed on 7 May 2021). In contrast, 41 motifs annotated with different functions were identified by motif search (https://www.genome.jp/tools/motif, accessed on 7 May 2021) (e.g., Porin_5). All *neuropeptide* genes exhibited the conserved structural domains of motif5 but multifarious functional domains. Based on the structural domains and functional domains, the *neuropeptide* genes were divided into different subfamilies, such as PBAN, FMRF, allatostatin, neuropeptide Y, and neuropeptide F. The PBAN (pheromone biosynthesis activating neuropeptide) family contains PBAN functional domains and a highly conserved domain (consisting of motif5, motif1 motif4, motif3, motif8, and motif2). The FMRF amide-related peptide family includes FARP functional domains and highly conserved residues (consisting of Phe-Met-Arg-Phe-NH_2_). The allatostatin family has allatostatin functional domains and highly conserved residues (consisting of Tyr/Phe-Xaa-Phe-Gly-Leu/Ile-NH2), and it could inhibit juvenile hormone biosynthesis by the corpora allata. Neuropeptide Y possesses PAH functional domains and a highly conserved domain (consisting of motif5, motif1, and motif6). Neuropeptide F harbors N_NLPC_P60 and kinocilin functional domains and a highly conserved domain (consisting of motif5, motif1, motif6, and motif7). Not only highly homologous sequences but also functional domains, such as PAH (pancreatic hormone domain, neuropeptide Y), were found in AXY04281.1 (the sequences of neuropeptide F2 in *S. exigua* downloaded from NCBI) and SeNPF. Therefore, these sequences were similar in function, and GO annotation showed that both were classified as neuropeptides and that *SeNPF* was classified as neuropeptide Y (Figure 6 and Supplementary Figure S5).

### 2.9. Analysis of the Effect of SeNPF Overexpression on Mating and Oviposition

**Figure 7:**
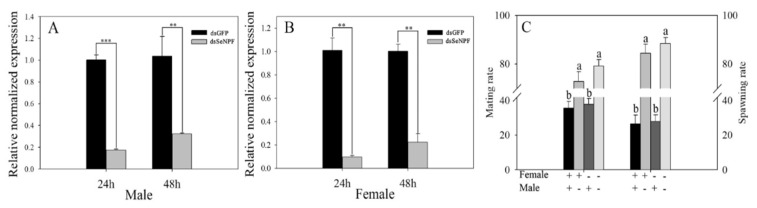
The effect of *SeNPF* on the mating and spawning rate in the SE-Sel strains. (**A**) The relative normalized expression of *SeNPF* of male adult after RNAi, (** means *p* < 0.01, *** means *p* < 0.001); (**B**) The relative normalized expression of *SeNPF* of female adult after RNAi, (** means *p* < 0.01); (**C**) The mating and spawning rate between different cross combinations (+ represents dsGFP, − represents dsSeNPF; e.g., the dark black (Female ‘+’, Male ‘+’) means that male injected with dsGFP and female injected with dsGFP are paired). Different numbers of asterisks indicate significant differences at different levels.

RNA interference is a very important means of molecular function verification, and the function of *SeNPF* of *S.* *exigua* was analyzed with this technology. After injection of dsSeNPF, the relative expression of *SeNPF* in the male adult of the SE-Sel strain was significantly downregulated (0.17- and 0.32-fold, respectively) (Figure 7A), and that in the female adult was also significantly downregulated at 24 h and 48 h (0.10- and 0.22-fold, respectively) (Figure 7B), compared with those fed by dsGFP. The mating rates of hybrid combination between the dsSeNPF male and the dsGFP or dsSeNPF female (72.92% and 79.17%, respectively) were significantly higher than those of the dsGPF male and the dsGFP or dsSeNPF female (35.67% and 37.83%, respectively), meaning that the oviposition rates of the dsSeNPF male and the dsGFP or dsSeNPF female were significantly increased by 84.51 and 88.50%, respectively (Figure 7C).

### 2.10. Statistics and Annotation of SNPs and Sequence Analysis of the RyR Genes

**Figure 8:**
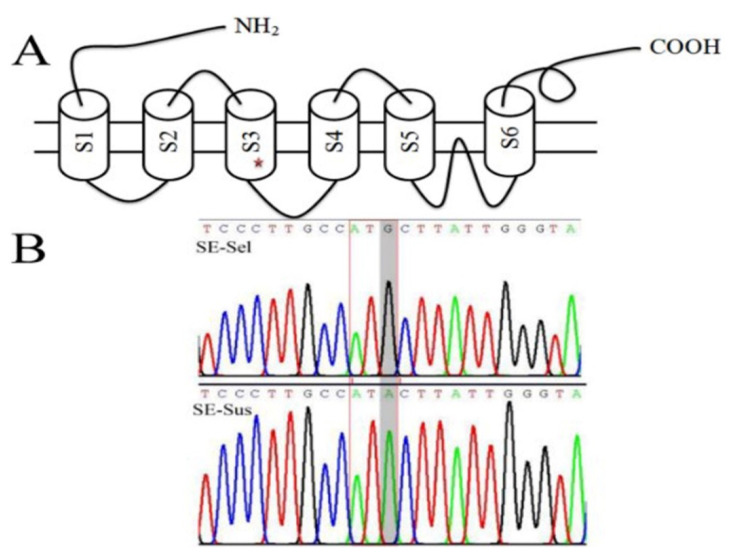
Peak map and structural analysis of cloned partial sequence of RyR in SE-Sel and SE-Sus strains. (**A**) The transmembrane domain topology; the red star is the location of the mutation; the positions of the transmembrane spanning domains S1 toS6 and based on those recently predicted for the diamondback moth RyR; [31,32] (**B**) Peak map; the box marked with black solid line indicates the mutation area, similarly hereinafter.

The mutation of RyR was analyzed by transcriptome and sequence cloning to explore the resistance mechanism of *S. exigua* to chlorantraniliprole and its fitness costs. Principal component analysis (PCA) and cluster dendrograms of the SNPs of six samples also showed that there was a significant clustering relationship in the samples of the SE-Sus strains (SE-Sus-1, SE-Sus-2, SE-Sus-3) and the SE-Sel strains (SE-Sel-1, SE-Sel-2, SE-Sel-3) (Supplementary Figure S6A and Supplementary Figure S6B). Additionally, the degree of the contribution of PC1 (60.3%) was significantly higher than that of PC2 (11.6%) (Supplementary Figure S6A). Some SNPs in *Unigene0007287* and *Unigene0010376* mapped to the specific amino acids 4631–4967 of RyR were identified through transcriptome data and sequence alignment between the SE-Sus and the SE-Sel strains. The sequence mapped to the specific amino acids 4631–4967 of RyR showed that I4743M (ATA-ATG), corresponding to I4790M in PxRyR, was found in the SE-Sel strain compared with the SE-Sus strain and no. JQ354988.1 in NCBI, and the I4743M mutation was located in the S3 transmembrane segments; although the secondary structure had not been changed, a branch at the mutation position had occurred (Figure 8 and Supplementary Figure S7).

## 3. Discussion

At present, the resistance of *S. exigua* to chlorantraniliprole is becoming increasingly serious. Fortunately, the development of insecticide resistance is commonly accompanied by fitness costs, along with the evolution of resistance [27]. Ribeiro et al. [24] revealed that the *P. xylostella* of the chlorantraniliprole-resistant field population presented a significantly lower larval weight and fecundity than the susceptible strain when not exposed, suggesting a fitness cost associated with resistance. When the resistant strain of *Nilaparvata lugens* was obtained by continuous selection in the presence of nitenpyram, the resistant strain showed a lower intrinsic rate of increase in the net reproductive rate, egg survival rate, and fecundity (eggs/female) compared to the susceptible strain [33]. However, both of the resistant strains ImiLabSel and ImiRes exhibited higher activities of cytochrome P450 monooxygenases by 72.3 and 40.5% and severe fitness costs (reductions of 86% for ImiLabSel and 68.0% for ImiRes) [34]. Our data also indicated that the ability of potential oviposited eggs and spawning rate per female of the SE-Sel strain were significantly decreased when successively exposed to chlorantraniliprole.

The formation and deposition of *Vg* is a fundamental step during the procedure of the vitellogenesis of insects, which is a prerequisite for the regulation of insect reproduction and the maturity of egg cell development [35], and it is traditionally considered a key factor in the female fertility of insects [36]. The decreased expression of *Vg* exerted negative impacts on the fecundity of *Chilo suppressalis* and *A. lucorum*, and there were significantly positive linear correlations between *SeVg* and *SeVgR* expression in *S. exigua* [37,38,39]. Our results demonstrated that the mRNA expression levels of *SeVg* and *SeVgR* were significantly reduced in the SE-Sel strain compared with the SE-Sus strain and were consistent with the number of potential oviposited eggs. Shu et al. [40] and Zhao et al. [36] also revealed that *Vg* was expressed specifically in the female fat body and was detectable after the fifth day of female pupation, then reached a maximum in 48-h-old or 36-h-old female adults and started to decrease quickly, which was similar to our results.

The *Vg* contents in the ovary depend not only on uptake by *VgR* into oocytes during vitellogenesis but also on its synthesis as affected by various elements, such as ecdysone and juvenile hormone (JH) [41,42]. Parthasarathy et al. [43] confirmed that JH directly regulated the synthesis of Vg in the fat body, and hibernation-triggered JH changes resulted in the upregulation of *Vg1* and *Vg2* and the production of queens [44]. There is a significant relationship among JH titers and the biosynthesis of insect JHs is regulated by juvenile hormone-binding proteins (transported to the target cells) [45] and neuropeptides, such as allatostatins [46] and allatotropins [47]. Neuropeptides also regulate the synthesis of Vg, and injections of corazonin peptide caused a decrease in *Vg* mRNA levels [48]. Our transcriptome data and RT-qPCR results also indicated that the expression levels of *SeNPF* and *SeNPFR* were significantly increased, even though those of the juvenile hormone-binding protein significantly decreased in the SE-Sel strain compared with the SE-Sus strain; this could explain why the expression levels of *SeVg* and *SeVgR* in the SE-Sel strain were low.

Except for the difference in potential oviposition, the spawning rate was also significantly lower because most of the eggs of the SE-Sel strain remained in the ovaries. Oviposition was stimulated after copulation, known as the post-mating responses (PMRs) in inseminated females, which was mediated by the (sex-peptide) SP/SPR (sex-peptide receptor) system [49,50]. PMRs require SPR in a limited number of sensory neurons in the female reproductive organ, and the knockdown of *SPR* in either fruitless or pickpocket-expressing neurons fails to induce PMR after mating [51]. However, the expression levels of *SeNPF* and *SeNPFR* in virgin adults of the SE-Sus strain were significantly increased compared with the SE-Sus strain. Then, neuropeptide F expression levels and intracellular Ca^2+^ activity (as an indicator of neural activation) in NPF neurons were altered by the animal’s mating status [52,53,54]. Liu et al. [55] found that knocking out *NPF*, a homolog of the mammalian neuropeptide Y, or suppressing the activity of NPF neurons reduced the inhibition of courtship behavior in sexually satiated males. Tuelher et al. [56] also found that chlorantraniliprole-treated males coupled with untreated females exhibited a higher number of mating events because the release and depletion of Ca^2+^ might affect the activity of NPF neurons. Phylogenetic tree analyses and gene molecular structure comparisons showed that there was high homology between neuropeptide F2 *AXY04281.1* and neuropeptide *SeNPF*. All neuropeptide F and neuropeptide Y family members in *Drosophila melanogaster* and *Spodoptera* insects had motif5, motif6, and motif1, but other neuropeptide families did not have a similar structure. Therefore, we speculate that the upregulation of neuropeptide *SeNPF* resulted in a lower probability of mating and spawning rate and may be the direct cause of the fitness cost of the SE-Sel strain. Gospocic et al. [48] found that the overexpression of the neuropeptide and neuropeptide receptor stimulated insect feeding and increased body weights in pupae and adults, similar to our results.

Our results of transcriptome analysis and cloning revealed the RyR^I4765M^ mutation in the SE-Sel strain. Mutation in RyR (such as T4826I) could cause a gain-of-function, i.e., Ca^2+^ leakage from the sarcoplasmic reticulum [57], which regulates neuropeptide activation [10,13,14]. Angela et al. found that some mutations (such as F4732D, G4733E, R4736W, and R4736Q) in the cytoplasmic loop between the S2 and S3 transmembrane segments would reduce Ca^2+^-dependent channel inactivation and result in the excessive release of calcium ions from the sarcoplasmic reticulum [58]; the RyR^I4743M^ mutation was located in the S3 transmembrane segments and might affect the release of calcium ions and the resistance and activity of NPF neurons. The mutations of the targets made it less susceptible to the action of the pesticide [59]. Chlorantraniliprole engaged critical residues within the C-terminal transmembrane assembly, particularly the amino acids 4610–4946 and 4631–4967 in *S. exigua*, according to BLAST analysis [60]. Silva et al. [61] found that the wicked evolution of RyR led to an increase in the resistance to chlorantraniliprole in the tomato pinworm *Tuta absoluta* rather than enhanced detoxification. Guo et al. [18,62] discovered that there was a significant correlation between the frequency of mutation (G4946E and I4790M) in RyR and the chlorantraniliprole resistance ratios in *P. xylostella*. The RyR^G4946E^ mutation introduced with CRISPR/Cas9 technology into the susceptible strain of *S. exigua* led to 223-fold resistance to chlorantraniliprole [63]. However, the RyR^G4946E^ mutation had not been detected in the SE-Sel strain. Roditaki et al. [64] and Zuo et al. [63] confirmed that the RyR^I4790M^ mutation also played an important role in the resistance to chlorantraniliprole. Therefore, the increase in the resistance to chlorantraniliprole in the SE-Sel strain, accompanied by the fitness cost, might be the result of the mutation of RyR, but how RyR mutation affects the upregulation of neuropeptides and induces a fitness cost requires further verification.

## 4. Methods and Methods

### 4.1. Insects

The resistance ratios for the SE-Sus and SE-Sel strains (with LC_50_ values of 0.502 μg/g and 5.837 μg/g, respectively) were estimated at 3.72- and 226.69-fold, compared with the LC_50_ value of 0.061 μg/g in Sus-Lab to chlorantraniliprole. All stages were maintained under the same standard conditions of 27 ± 1 °C, 70–80% RH, and a 16:8 h (L:D) photoperiod. The larvae and adults were reared with an artificial diet and a 10% sugar solution, respectively. Pupae and newly laid eggs were sterilized with a 0.2–0.3% sodium hypochlorite disinfectant solution (Wang et al., 2018). The susceptible strain (Sus-Lab) of *S. exigua* was alimented in the laboratory without exposure to any insecticide since 2003. Leshan’s population from the original field samples was collected in 2017 from vegetable fields in the Mouzi town of Leshan City in Sichuan Province, China. The SE-Sus strain was established from the untreated offspring of pairs in the Leshan population with the highest mortality by continual selection with gradually increasing concentrations of chlorantraniliprole, on account of the LC_70_ values from the bioassay of their parent generations for a total of 6 generations. In contrast, SE-Sel was screened by treating the offspring of pairs with gradually diminishing concentrations of chlorantraniliprole, on account of the LC_30_ values for a total of 6 generations, according to the method described by Huang et al. [65]. The resistance ratios for the SE-Sus and SE-Sel strains (with LC_50_ values of 0.502 μg/g and 5.837 μg/g, respectively) were estimated at 3.72- and 226.69-fold, compared with the LC_50_ value of 0.061 μg/g in Sus-Lab to chlorantraniliprole. All stages were maintained under the same standard conditions of 27 ± 1 °C, 70–80% RH, and a 16:8 h (L:D) photoperiod. The larvae and adults were reared with an artificial diet and a 10% sugar solution (*w*/*w*), respectively. Pupae and newly laid eggs were sterilized with a 0.2–0.3% sodium hypochlorite disinfectant solution (*w*/*w*) [20].

### 4.2. Insecticide and Reagents

Chlorantraniliprole (95%) was obtained from DuPont Agricultural Chemicals Co., Ltd. (Wilmington, DE, USA). Triton X-100 was obtained from Amresco Co. (Solon, TX, USA). All other chemicals, solvents, and analytical-grade reagents were obtained from Chengdu Aikeda Chemical Reagent Co., Ltd. (Chengdu, China).

### 4.3. Mating Behavior and Reproductive Bioassays

Eight pairs of virgin female and male couples (3 days after emergence) of the SE-Sus and SE-Sel strains were placed in the mating cup (glass tubes containing 10% honey water (*w*/*w*)) to be allowed to mate; the female ovaries after mating 24 h were dissected, and if there were spermatophores, the mating was considered successful, and the mating ratios (percentage of successful mating couples) were recorded [56]. The newly hatched larvae were raised to pupae in glass tubes (diameter × height: 2.0 cm × 8.0 cm) containing an artificial diet. The pupae were soaked in a 0.5% sodium hypochlorite solution (*w*/*w*) and disinfected. Three pairs of male and female of the SE-Sus strain or SE-Sel strains, respectively, were placed in a transparent plastic cup with a lid. Each treatment was repeated eight times, and 10% honey water (*w*/*w*) was provided to nourish their eggs. The plastic cups and honey water were replaced every 24 h, the eggs were collected, and the number of oviposited eggs per head (designated a) was recorded until the end of oviposition. During the peak period of oviposition, 10 eggs were randomly selected. After hatching, the number of hatched larvae (designated k) and the number of unhatched larvae (designated m) were counted. After the female adult died, the ovaries were dissected under an anatomical microscope to record the number of eggs left in the ovarian duct (considered non-oviposited eggs, b). The potential oviposited eggs (designated n), oviposition rate, and hatchability were calculated by the following formulas: potential oviposited eggs = a + b; oviposition rate=[a/n]×100%; and hatchability=[ k∕(k +m)]×100%.

### 4.4. Ovary Solution Plane

Forty SE-Sus and SE-Sel strains at the pupal stage were collected and separated with finger tubes, and the weight of each pupa was recorded with an electronic balance after 24 h of pupation. After 24 h of emergence, the female adults were weighed (designated c) under CO_2_ anesthesia, washed in clear water, and put into phosphate-buffered saline. The abdominal epidermis of the adults was torn with two tweezers to expose the reproductive organs. The fat body and epidermis were removed as far as possible, the ovaries were gently pulled out, and the ovarian tubes were gently moved with an anatomical needle to fully expand the tubes. The length of the ovarian tubes was measured, and the absorbent paper was removed. The fresh weight of the ovary (designated d) was measured and recorded. The ovarian index was calculated using the following formula: Ovarian  index=(c∕d)×100%.

### 4.5. Gene Expression Pattern of SeVg in the SE-Sus and SE-Sel Strains

The SE-Sus and SE-Sel strains were fed in the finger tubes. When the larvae reached the prepupal stage, they were transferred to a culture plate with artificial feed cells. Ten female pupae, from the 1st to the 8th day of pupation, and female adults emerged for 0, 12, 24, 36, 48, 60, and 72 h, were collected. The ovaries and fat bodies were obtained from the dissected pupae and dissected female adults, loaded into 2 mL centrifugal tubes, instantly quick-frozen in liquid nitrogen, and stored at −80 °C. RNA extraction, reverse transcription, and qPCR of the *SeVg* gene were performed in accordance with the procedure described below.

### 4.6. Transcriptome Analysis

In accordance with the manufacturer’s instruction for the TRIzol^®^ Reagent (Invitrogen™, ThermoFisher Scientific, Waltham, MA, USA), approximately 10 ovaries and fat bodies of virgin adults emerged for 36 h from the SE-Sus and SE-Sel strains were used for total RNA isolation. cDNA library construction and sequencing, splicing, and unigene annotation, covering Nr annotation, KEGG annotation, COG/KOG functional annotation, and Gene Ontology (GO) annotation, were performed in sequence according to the method described by Wang et al. [66]

The expression level of unigenes was determined by the RPKM method, according to the following formula: RPKM (A) = (1,000,000*C)/(N*L/1000) [67]. The RPKM (A) value denotes the expression level of gene A; the C value denotes the number of reads that uniquely mapped to gene A; the N value denotes the total number of reads that uniquely mapped to all genes, and the L value denotes the number of nucleic acid bases in gene A.

The dramatically differentially expressed genes (DEGs) between the SE-Sus and SE-Sel strains were screened by edge R. The screening threshold values were FDR < 0.05 (*p*-value after calibration by FDR) and |log2FC| > 1, and GO and KEGG enrichment analysis were performed for the DEGs.

### 4.7. Quantitative PCR (qRT-PCR)

Total RNA of the ovaries and fat bodies of virgin adults emerged for 36 h from the SE-Sus and SE-Sel strains was extracted and reverse-transcribed according to the procedure described in Section 4.6.

The cDNAs of *Vg* (*KT599434.1*), two juvenile hormone-binding proteins (*SeJHBWDS3* and *SeJHBAN*), a vitellogenin receptor (*SeVgR*), a neuropeptide *SeNPF*, a neuropeptide receptor *SeNPFR,* and the *F-actin* gene [66] from the SE-Sus and SE-Sel strains were amplified by PCR with twelve pairs of corresponding primers (Table 1). The system and procedure of RT-qPCR were performed according to the method described by Wang et al. [66].

### 4.8. Diversity and Collinearity of Neuropeptide Genes

A total of 63 complete neuropeptide amino acid sequences of *Drosophila melanogaster* and *Spodoptera* insects were downloaded from the NCBI database and analyzed for the conserved functional domains with the motif search (https://www.genome.jp/tools/motif, accessed on 7 May 2021) and meme (http://meme-suite.org/tools/meme, accessed on 7 May 2021) tools. The results were visualized with TBtools software.

### 4.9. Statistics and Annotation of Single Erroneously Paired Nucleotides and Sequence Analysis of RyR Genes

Based on the alignment results between the reads of the two treatments and the assembled unigenes database used by TopHat2, single erroneously paired nucleotides (SNPs), as determined by comparison with the assembled unigenes, were distinguished by The Genome Analysis Toolkit (GATK) [68]; then, these SNP sites were analyzed by the program SnpEff [69]. Furthermore, it was possible to analyze whether these SNP sites affected the gene expression level or encoded protein type. On the basis of the cDNA fragments (*Unigene0007287* and *Unigene0010376*) of RyR from RNA-seq data compared with ALL55470.1 in NCBI, we cloned the sequence containing the specific amino acids 4631–4967 of RyR. The cDNAs of RyR genes were amplified by PCR with corresponding primer pairs (Table 2). TransStart^®^ FastPfu DNA Polymerase (TransGen Biotech, Beijing, China) was used in the PCR. PCR was conducted with a PCR cycle of 95 °C for 2 min, 35 cycles of 95 °C for 20 s, 60 °C for 20 s, and 72 °C for 1 min, ending with an extension at 72 °C for 5 min. Amplified fragments were purified by a TIANgel^®^ Midi Purification Kit (Tiangen, Beijing, China) and sequenced at Shanghai Biological Engineering Co., Ltd. (Shanghai, China).

### 4.10. RNAi

A 241 bp PCR fragment of *SeNPF* with a homologous sequence of L4440 was amplified by the corresponding primer pairs (Table 2), and then cloned into the linearized vector L4440, amplified by the corresponding primer pairs, by the Cloning Kit (ClonExpress^®^II One Step Cloning Kit, Novoprotein Sicientific Inc., Shanghai, China). L4440-*SeNPF* was transformed into HT115 (DE3) and expressed according to the method described by Ma et al. [70]. Control treatments were prepared in the same way but mixed with bacteria transformed with the L4440-GFP vector. Extraction and purification of dsSeNPF and dsGFP were performed by using Zymoclean Gel RNA Recovery Kit (Zymo™, Jiangsu Hongqi Biological Technology Co., Ltd., Nanjing, China) according to the manufacturer’s instructions. After the determination of their integrity, 50 nL (approximately 150 ng) dsRNA was injected into the conjunction between the prothorax and mesothorax of freshly emerging female and male adults of the SE-Sel strain using a Micro 4TM microinjection device (MicroSyringe Pump Controller, World Precision Instruments, Hertfordshire, UK), according to the method described by Ruan et al. [71]. Their mating ratio and oviposition rates were recorded.

### 4.11. Data Analysis

The relative normalized expression of the screened genes and validated genes, the number of oviposited eggs, non-oviposited eggs, hatched and unhatched larvae, the weights of pupae, female adults and organs, and the length and number of the ovarian tubes in the SE-Sus and SE-Sel strains were compared using analysis of variance (ANOVA) followed by Tukey’s test for multiple comparisons (*p* < 0.05) with the SPSS version 17.0 software package (IBM).

## 5. Conclusions

On the basis of our results, we also found that the fitness costs were accompanied by the development of insecticide resistance; for example, the potential oviposition and the oviposition rate of the SE-Sel strain were significantly lower than those of the SE-Sus strain because the expression of *SeVg* and *SeVgR* in the SE-Sel strain was always lower than in the SE-Sus strain; as a post-mating response, the higher nonoviposition in the SE-Sel strain was caused by the lower mating rate. Moreover, the RyR mutation could affect the activities of neuropeptides; it led to the upregulated expression of the neuropeptide *SeNPF* and its receptor, *SeNPFR*. The mating and oviposition rate were significantly recovered when the *SeNPF* was knocked down though RNA interference (RNAi) in the male adult of the SE-Sel strain. Moreover, the neuropeptide *SeNPF* could influence the expression of *juvenile hormone-binding protein*, and it led to the downregulated expression of *SeVg*. Therefore, these results indicate that the fitness cost accompanied by chlorantraniliprole resistance in *S. exigua* may be related to the decrease in mating desire due to *SeNPF* overexpression.

## Figures and Tables

**Table 1:** Primers of differentially expressed genes for RT-qPCR.

Gene	Primer	Sequence (5′–3′)	Length
*CYP6AEW*	4495-F	AAAAAGAATGTACCGCACGCC	148
	4495-R	CCGTTCCGTAGTAAGCTCCA	
*SeABCG23*	6176-F	TGGAAGCTACGGAGCACTTG	124
	6176-R	GGTCCAGCGAAGTCAGTCAA	
*Vitellogenin receptor*	SeVgR-F	CCCAGGAAGAGAATGTTAGCGA	116
	SeVgR-R	GGTAGATACCTTGAGGGGTGC	
*SeABCOK*	15443-F	GCTGGTAGTATGCTCGGTCC	106
	15443-R	TTCAATGGTCCCGTGGAAGG	
*SeJHBWDS3*	16076-F	CTAGTAACGCAAGCCACGGT	145
	16076-R	GAACTTCGGTGCCAACCTCT	
*SeJHBAN*	18636-F	CAAGAACCCATCCGAAGCCT	149
	18636-R	CCACAGCCTCGTCTTTGTCT	
*SeGST15*	19590-F	CCCGGAGGTGTAGCCAAAAT	169
	19590-R	TGTTGGGGTACGTTGCTTCA	
*SeGST1*	20364-F	CCGTGCCATCAGCAGATACT	182
	20364-R	CATCGTCAGCTTTTGCTCCG	
*SeGSTZ2*	21647-F	AGGGAACTCTGTGAGGTGGT	124
	21647-R	TTAAGCCACGGTCAGTCCAG	
*SeCarEs1*	27390-F	TCGATGTGCTCGGCTTTCTT	120
	27390-R	GGTCACCACCGAAGTTAGCA	
*SeCYP6AB10*	34483-F	TTCATCGGTGTCTGCGCATT	131
	34483-R	ATAATCTTCAGGGCACCGGC	
*SeNPFR*	35241-F	TAGGCGAGGCTTCAAACAGG	132
	35241-R	CGCCGCGTCATACCATTTAC	
*SeNPF*	38343-F	GAACGTTTCGACACTGCTGA	130
	38343-R	CTCTGAAGATCACGGAGGCA	
*Vitellogenin*	Vg-F	CACTCTGCCGTATCTCGCAT	130
	Vg-R	GTTGAACGTGGCTGTGAACC	
*Actin*	Actin-F	AGGGAAATCGTGCGTGACAT	120
	Actin-R	GACCGTCGGGAAGTTCGTAG	

**Table 2:** Primers of partial *RyR* for clone.

Gene	Primer	Sequence (5′–3′)
*RyR*	RyRs-F	GCAAGCTCAAGAGCGTATGG
	RyRs-R	CGGTAGACCTCGGAGTCATC

## Data Availability

The raw RNA-seq data have been deposited in the NCBI sequence read archive (BioProject ID: PRJNA587951).

## Supplementary Materials

The following are available online at https://www.mdpi.com/article/10.3390/ijms22095027/s1.
