# Supplementary material for: Fitness Costs of Chlorantraniliprole Resistance Related to the SeNPF Overexpression in the Spodoptera exigua (Lepidoptera: Noctuidae)
Source: Int J Mol Sci. 2021 May 10;22(9):5027. doi: 10.3390/ijms22095027 (PMC8126017; doi:10.3390/ijms22095027)
Supplement: Supplementary file 1 [file ijms-22-05027-s001.zip › ijms-1181601-supplementary.pdf]

Supplementary table 1. Statistics of transcriptome data filtering

Sample	Before Filter Reads Num	After Filter clean Reads Num	GC	Adapter (%)	Low quality(%)
**SE-Sus1**	42072370 (Q20:98.16%, Q30:95.42%)	41227408 (Q20:98.91%, Q30:96.65%)	46.14 %	196598 (0.47%)	631060 (1.5%)
**SE-Sus2**	51630904 (Q20:98.19%, Q30:95.46%)	50638352 (Q20:98.91%, Q30:96.65%)	46.05 %	221600 (0.43%)	750690 (1.45%)
**SE-Sus3**	50278778 (Q20:98.17%, Q30:95.44%)	49286680 (Q20:98.91%, Q30:96.66%)	45.87 %	223220 (0.44%)	748588 (1.49%)
**SE-Sel1**	49571700 (Q20:98.14%, Q30:95.39%)	48556562 (Q20:98.91%, Q30:96.64%)	46.03 %	232782 (0.47%)	762250 (1.54%)
**SE-Sel2**	51661964 (Q20:98.20%, Q30:95.50%)	50656250 (Q20:98.92%, Q30:96.70%)	45.55 %	219522 (0.42%)	765324 (1.48%)
**SE-Sel3**	51474908 (Q20:98.16%, Q30:95.43%)	50444866 (Q20:98.91%, Q30:96.66%)	45.86 %	230874 (0.45%)	778440 (1.51%)

Note: Reads Num (PE) = Reads1 Num + Reads2 Num (the reverse and forward total reads); the probability of error recognition of Q20 and Q30 were 1% and 0.1%, respectively.

Supplementary table 2. Partial differential genes annotated

Annotation	GeneID	log2 Ratio(K/M)	Pvalue	Gene type
Juvenile hormone binding protein	*Unigene0016076*	-3.5825	5.27E-199	*SeJHBWDS3*
	Unigene0018681	-1.2038	1.08E-16	*SeJHBAN*
Vitellogenin receptor	*Unigene0012209*	-4.4602	4.09E-10	*SeVgR*
Neuropeptide	*Unigene0038343*	3.389	0.0001277	*SeNPF*
Neuropeptide receptor	*Unigene0035241*	12.531	6.28E-206	*SeNPFR*

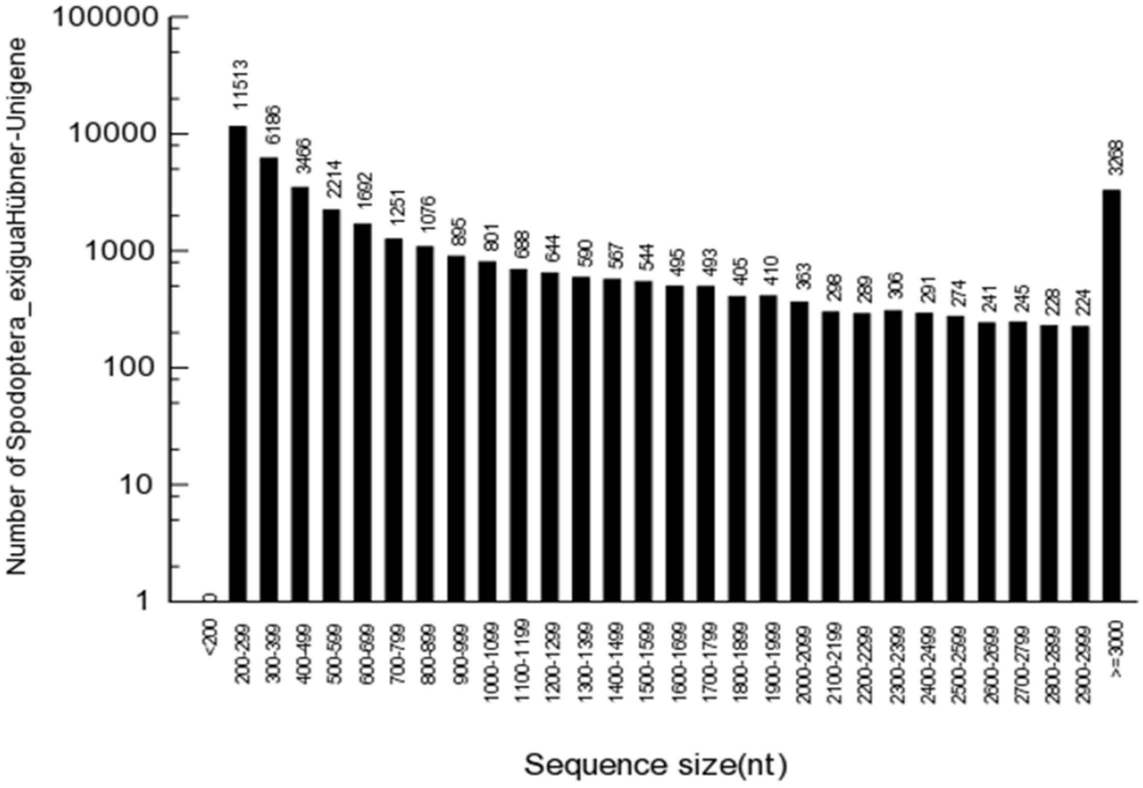

Supplementary figure 1a. Distribution of unigenes in different length ranges in the transcriptome data *of S. furcifera* reads and coverage map of reads in unigene (e.g.,<200 represents 0–200).

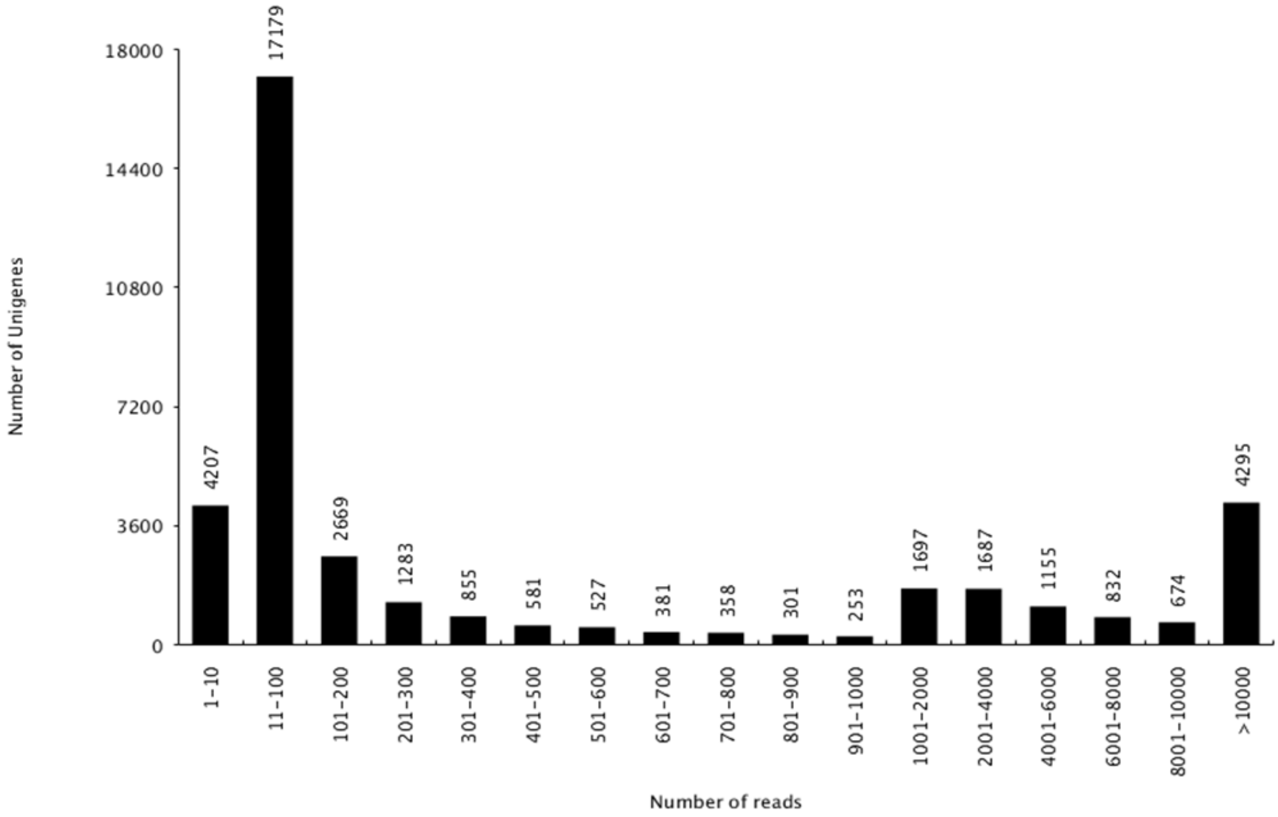

Supplementary figure 1b. Distribution of unigenes with different number of reads in the transcriptome data *of S. furcifera* reads and coverage map of reads in unigene (e.g.,1-10 represents unigene consisted with 1-10 reads).

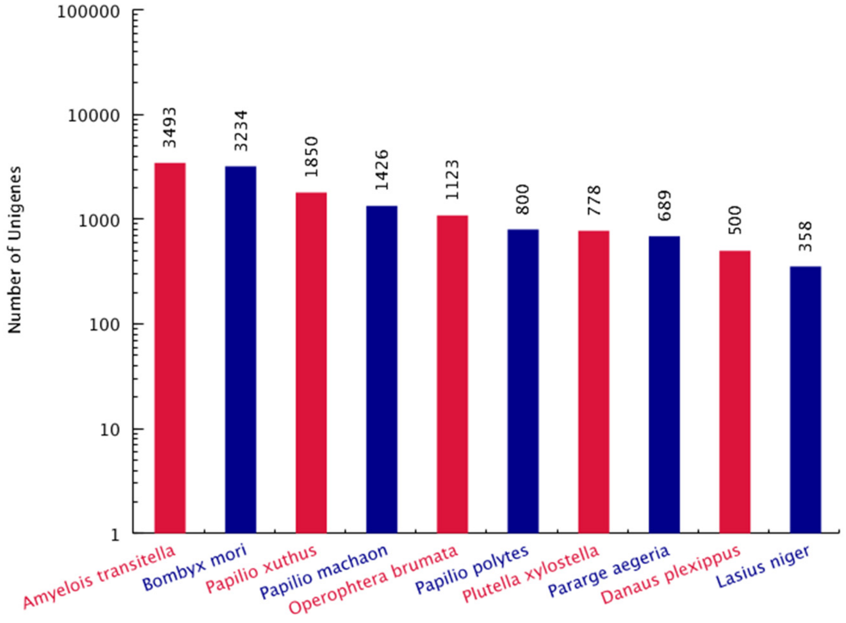

Supplementary figure 2a. Species distribution of the BlastX matchs to the unigene on the Nr database (Top ten)

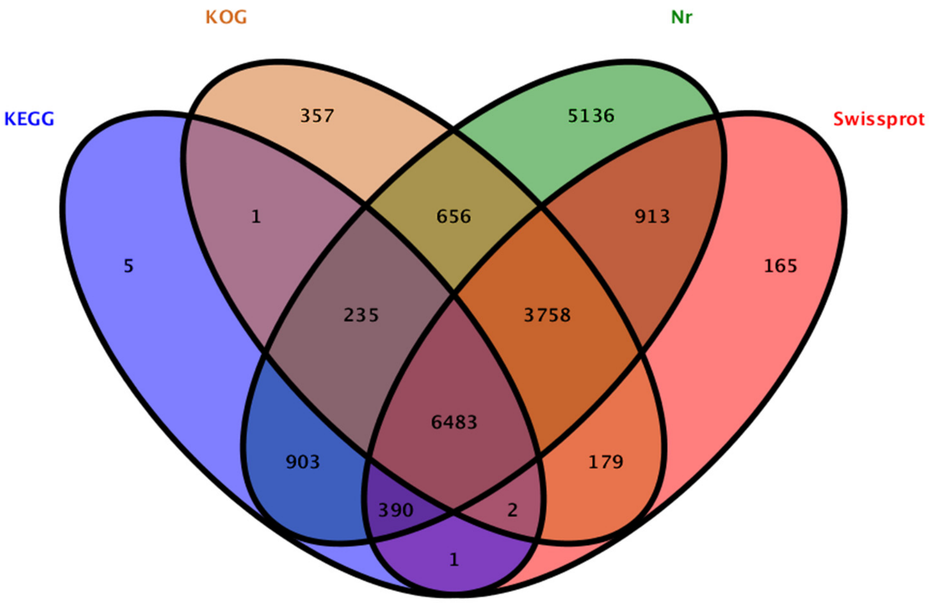

Supplementary figure 2b. Venn Diagram of annotation genes of four data base

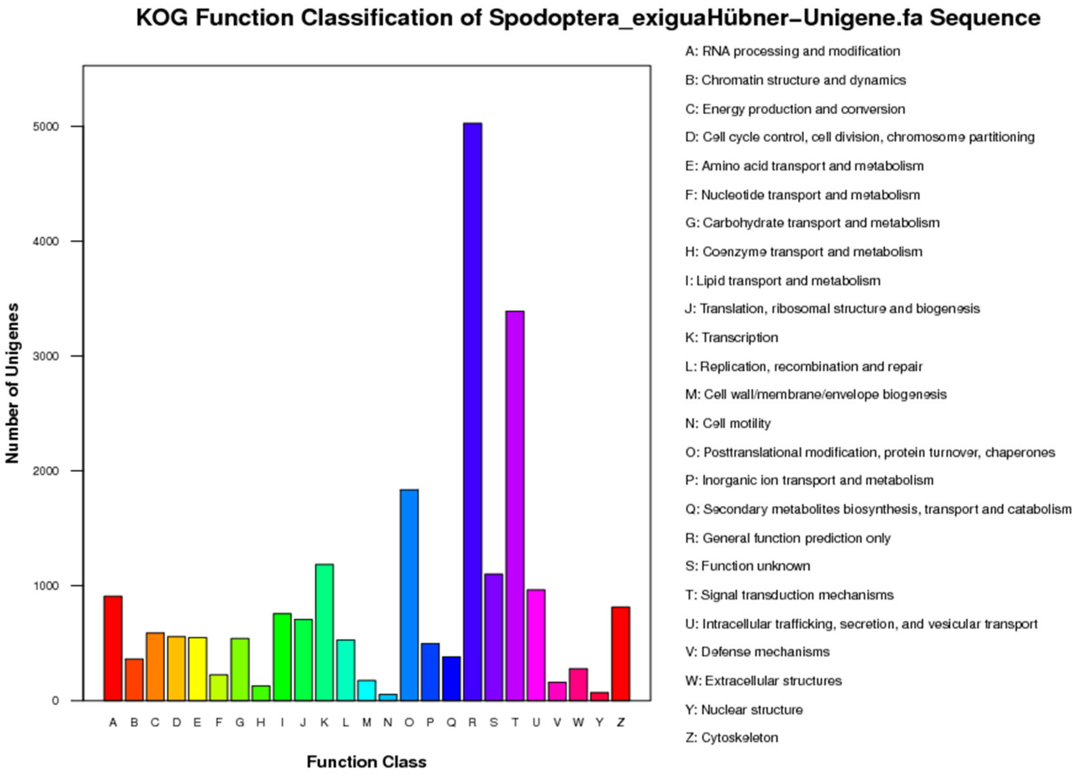

Supplementary figure 2c. KOG function classification chart

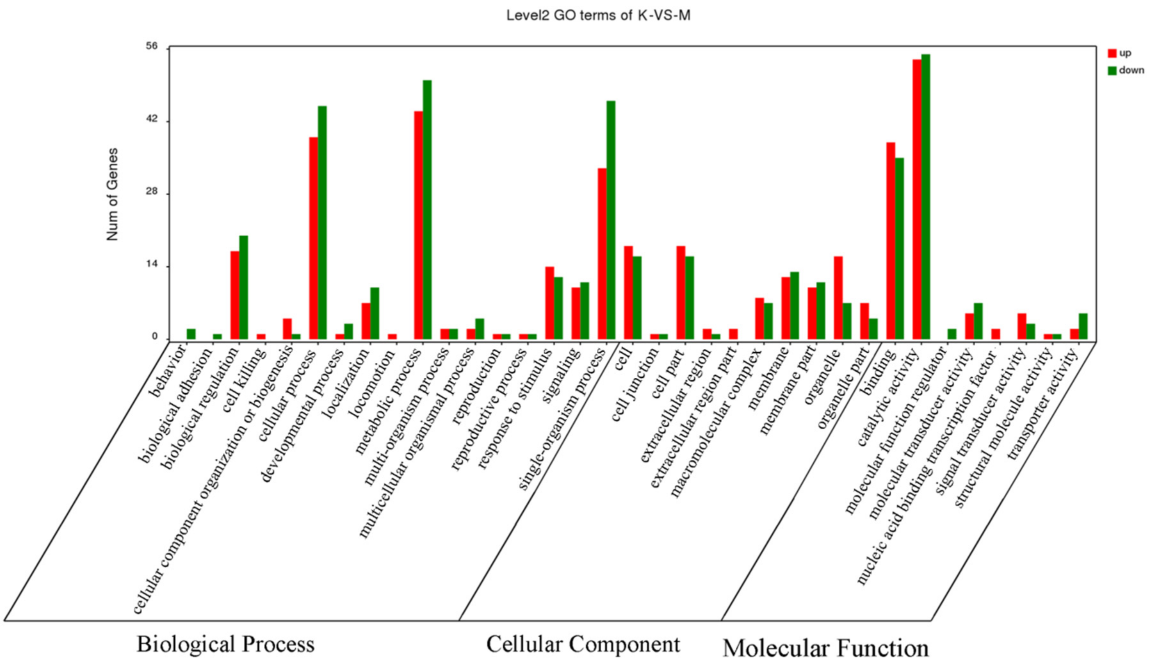

Supplementary figure 3. GO categories of unigene on the different groups. The ordinate means GO terms, the abscissa means the number of DEGs of each GO term. Red color means the terms of up-regulated genes, green color means the terms of down regulated genes, respectively.

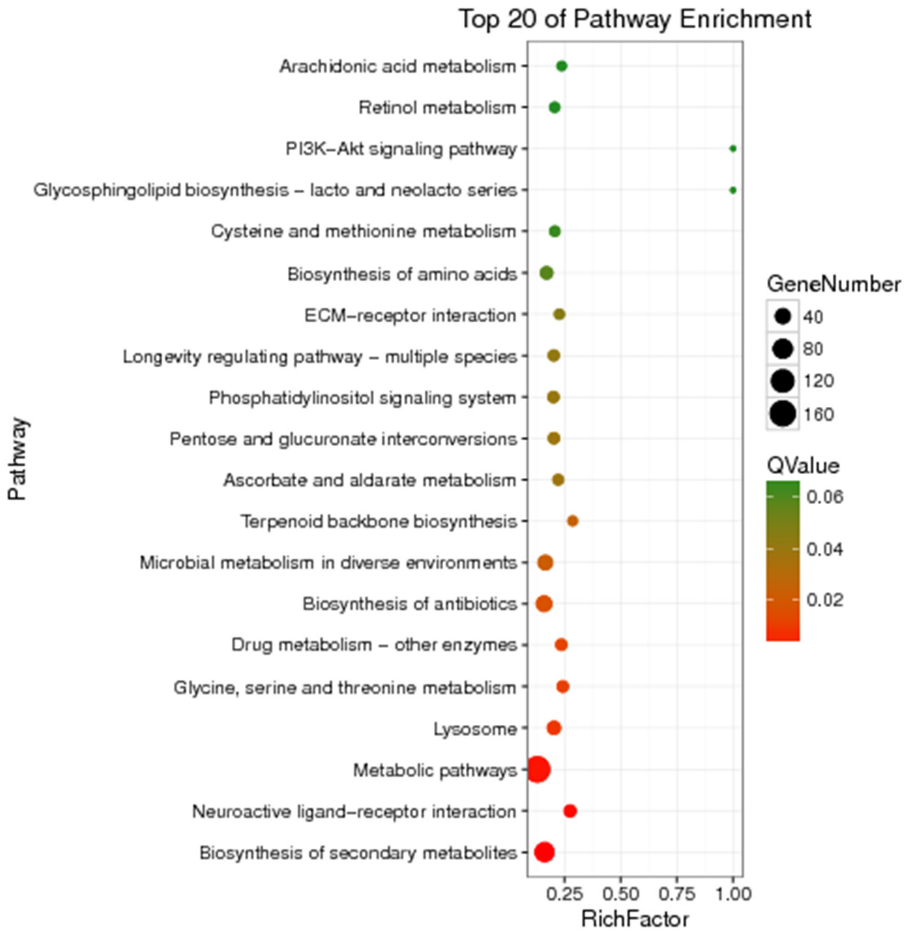

Supplementary figure 4. Bubble diagram of pathway enrichment of unigene on the different groups (Top 20). The abscissa means KEGG terms, and the ordinate means rich factor of each term. Red color means the terms of significant enrichment, and the bubble size indicates the number of enriched genes.

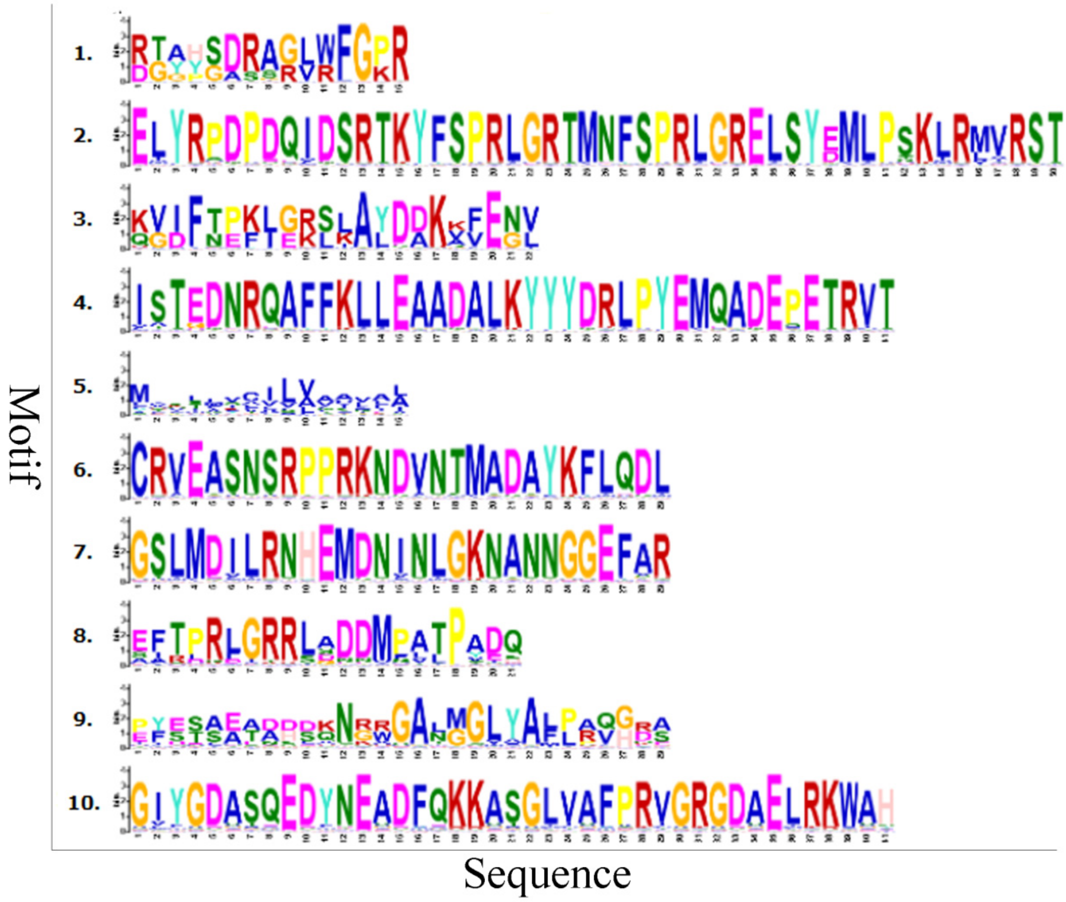

Supplementary figure 5. Conservative sequence display of different motif structures. The number of 1 to 10 stands for means the identifier of different motif structures, and abscissa represents conserved amino acid sequences of different motifs.

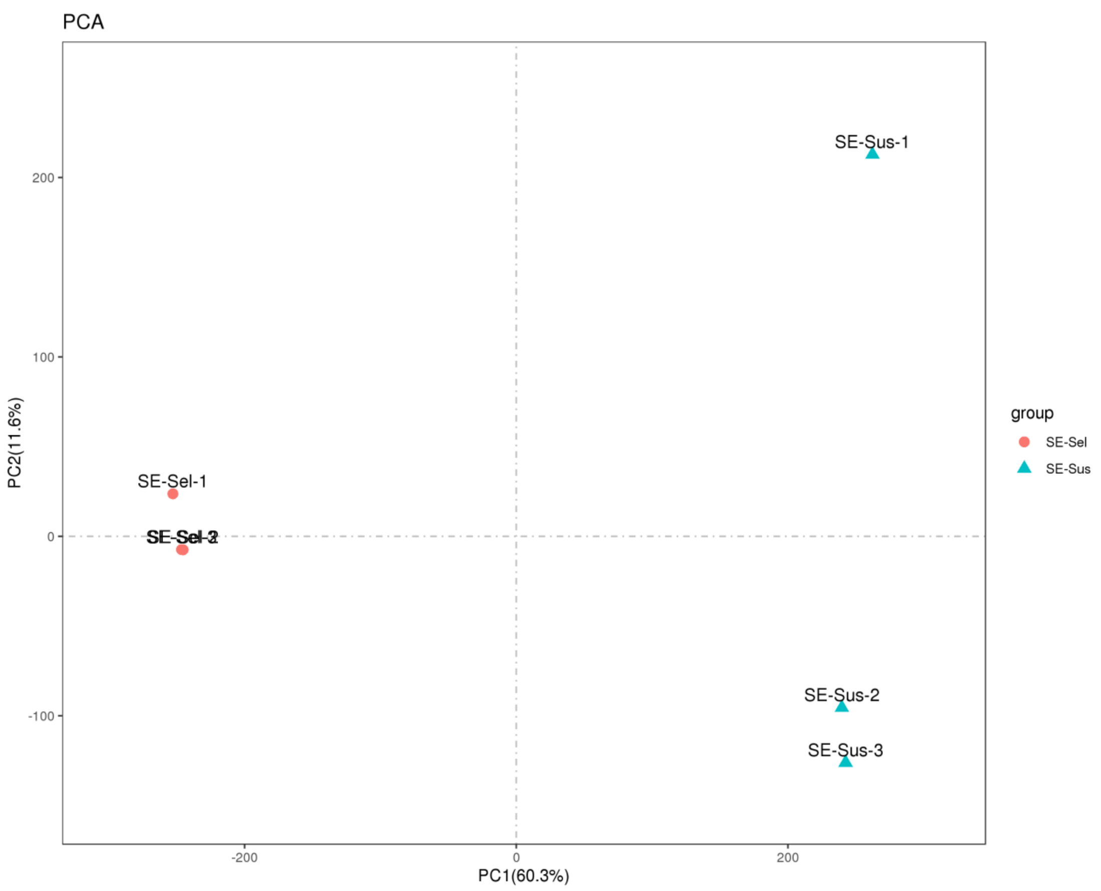

Supplementary figure 6a. Principal component analysis (PCA) of the Single Erroneously Paired Nucleotides (SNPs) of six samples

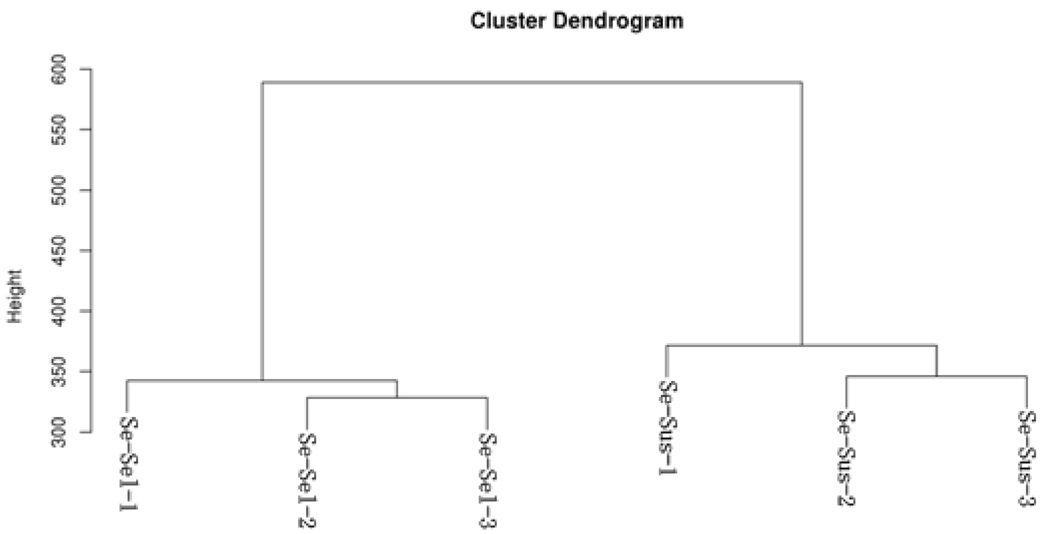

Supplementary figure 6b. Cluster dendrogram of the Single Erroneously Paired Nucleotides (SNPs) of six samples

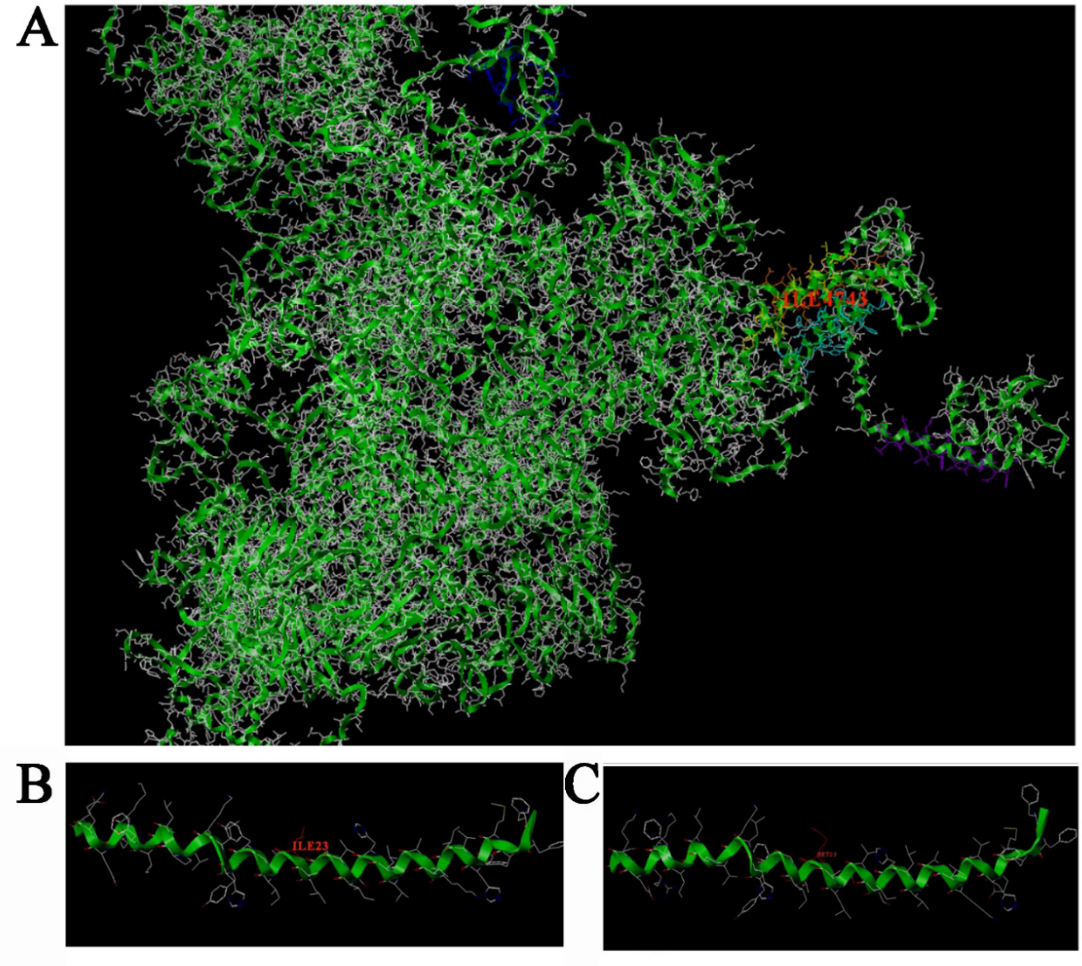

Supplementary figure 7. A—Three-dimensional structure, yellow is S3, blue is S1, organge is S2, cyan is S4, violet is S5, template is the type 2 ryanodine receptor ryr2 (5goa.1), [32] with a 47.91% homology; B and C—The secondary structure of the sequence nearby the S3 transmembrane domain.
